# Isolation, Identification and Investigation of Fermentative Bacteria from Sea Bass (*Dicentrarchus labrax*): Evaluation of Antifungal Activity of Fermented Fish Meat and By-Products Broths

**DOI:** 10.3390/foods9050576

**Published:** 2020-05-04

**Authors:** Francisco J. Martí-Quijal, Andrea Príncep, Adrián Tornos, Carlos Luz, Giuseppe Meca, Paola Tedeschi, María-José Ruiz, Francisco J. Barba, Jordi Mañes

**Affiliations:** 1 Nutrition, Food Science and Toxicology Department, Faculty of Pharmacy, Universitat de València, Avda. Vicent Andrés Estellés, s/n, 46100 Burjassot, València, Spain; francisco.j.marti@uv.es (F.J.M.-Q.); anprince@alumni.uv.es (A.P.); tornos@alumni.uv.es (A.T.); carlos.luz@uv.es (C.L.); giuseppe.meca@uv.es (G.M.); m.jose.ruiz@uv.es (M.-J.R.); jordi.manes@uv.es (J.M.); 2 Department of Chemical and Pharmaceutical Sciences, University of Ferrara, Via Fossato di Mortara 17, 44121 Ferrara, Italy; tdspla@unife.it

**Keywords:** sea bass, by-products, fermentative process, antifungal activity, lactic acid bacteria

## Abstract

During fish production processes, great amounts of by-products are generated, representing ≈30–70% of the initial weight. Thus, this research study is investigating 30 lactic acid bacteria (LAB) derived from the sea bass gastrointestinal tract, for anti-fungal activity. It has been previously suggested that LAB showing high proteolitic activity are the most suitable candidates for such an investigation. The isolation was made using a MRS (Man Rogosa Sharpe) broth cultivation medium at 37 ºC under anaerobiosis conditions, while the evaluation of the enzymatic activity was made using the API^®^ ZYM kit. Taking into account the selected bacteria, a growing research was made fermenting two kinds of broths: (i) by-products (WB), and (ii) meat (MB). Both were fermented at three different times (24, 48 and 72 h). Then, the antifungal activities of both fermented by-products and meat broths were determined qualitatively and quantitatively in solid and liquid medium against two different strains of the genera *Penicillium*, *Aspergillus* and *Fusarium*. After the experiments, a total of 30 colonies were isolated, observing a proteolytic activity in 7 of the isolated strains, which belong to *Lactobacillus* genus, and the two more active strains were identified by polymerase chain reaction (PCR) as *L. plantarum*. Several strains evidenced antifungal activity showing an inhibition halo and Minimum Inhibitory Concentration (MIC) and Minimum Fungicidal Concentration (MFC) values between 1–32 g/L and 8–32 g/L, respectively. In conclusion, the isolated bacteria of sea bass had the ability to promote the antifungal activity after the fermentation process, thus being a useful tool to give an added value to fish industry by-products.

## 1. Introduction

Nowadays, the increased fish production, up to 171 million tons in 2016 [1], is also generating an increase in food waste and by-products, which can represent from 30% to 70% of the total weight of the fish [2].

Among the main by-products obtained from fish, eyes (0.8–1.5%), skin (1–3%), head (9–12%), viscera (12–18%), spines (9–15%) and muscle discards (40–55%) are the most representative [3], all of them having an important content of nutrients and bioactive compounds. For the fish industry, meat constitutes the main useful product, accounting for more than 50% of the fish. However, there are several potential uses for the inedible parts, including skin, viscera, heads and cartilage. Therefore, the industry has been forced to propose several options for the valorization of these by-products in order to develop an integral and sustainable use of fish [4,5].

Thus, taking into account the current trend regarding the increased production of high-added-value compounds from natural sources, which can control biological contamination, fish by-products can constitute a useful tool [6]. In this sense, the use of fermentation can be an interesting strategy to recover the valuable compounds from fish by-products [7]. Fermentation is a conventional process used since ancient times for food preservation, however, it has not been traditionally used to recover high-added-value compounds from fish by-products, although it presents a clear potential for this purpose [8]. For instance, some previous research evaluated lactic acid fermentation produced by lactic acid bacteria (LAB) to recover biomolecules from different by-products, observing its great potential to extract oil [9,10,11,12], chitin [13,14], proteins and antimicrobial and antioxidant compounds from fish by-products [7,9,15]. However, at this stage of development, there are no studies evaluating the effect of fermentation assisted by LAB to recover compounds from sea bass by-products with an antifungal activity. One of the factors to take into account to maximize the recovery of compounds is the isolation and use of bacteria belonging to the targeted fish, as using microorganisms other than the ones belonging to the natural microbial flora of the fish would cause non-desired alterations at the end products.

For instance, microorganisms isolated from sea bass viscera or metabolic products from these microorganisms could be used in order to inhibit growth or eliminate unwanted biological contamination, thus increasing food safety and product shelf life [16]. In this sense, other previous studies have confirmed the use of LAB, especially *Lactobacillus plantarum,* as the most relevant antifungal species in food, being an alternative to conventional preservatives, and showing an important inhibition of different fungi of genera *Aspergillus*, *Penicillium* and *Rhizopus*, among others [17]. 

**Figure 1:**
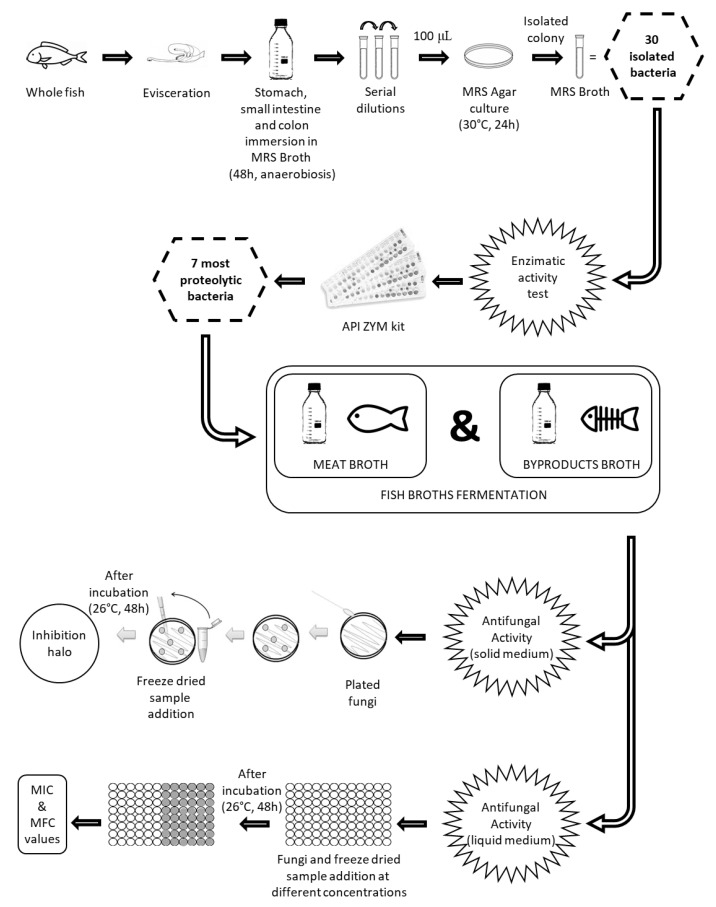
Schematic representation of the experimental set-up. MRS: Man, Rogosa & Sharp; MIC: Minimum Inhibitory Concentration; MFC: Minimum Fungicidal Concentration.

In this sense, as it is shown in Figure 1, the general objective of the present study was to screen for the enzymatic activity of lactic acid bacteria strains isolated from sea bass gastrointestinal tract in order to select the most proteolytic ones. These were then used to ferment by-products from sea bass to obtain compounds endowed with antifungal activity.

## 2. Materials and Methods 

### 2.1. Materials

#### 2.1.1. Samples

Fresh sea bass (*Dicentrarchus labrax*) samples (whole fish) fished from the Mediterranean Sea were bought at a local market in Tarragona, Spain. They were kept fresh at a temperature of 4 °C until dissection.

#### 2.1.2. Microorganisms

A total of 30 bacteria were isolated from sea bass (sixteen from the colon, seven from the intestine and seven from the stomach). To carry out the different tests, we selected the seven bacteria with the highest proteolytic activity [18] (two from the colon, one from the intestine and four from the stomach).

In order to evaluate the antifungal activity, strains of *Aspergillus parasiticus* CECT 2681, *Penicillium expansum* CECT 2278 and *Penicillium flavus* CECT 2949 were obtained from the Spanish Type Culture Collection (CECT Valencia, Spain). *Fusarium graminearum* ITEM 126 and *Fusarium verticillioides* ITEM 1205 strains were obtained from the Agro-Food Microbial Culture Collection of the Institute of Sciences of Food Production (Bari, Italy). The fungus *Penicillium verrucosum* VTT D 01847 was obtained at the VTT Technical Research Center of Finland (Turku, Finland).

#### 2.1.3. Consumables

The culture media used consisted of MRS (Man Rogosa Sharpe) Broth, MRS Agar, PDA (Potato Dextrose Agar) and PDB (Potato Dextrose Broth). They were purchased from Liofilchem Bacteriology Products (Roseto, Italy). The anaerobic microbiology incubation system (Anaerobic^®^) was obtained from Merck (Darmstdt, Germany). For the Gram test, the Gram Color Kit containing Crystal Violet, Lugol PVP, Safranina and Decolorizing solution was used, and it was purchased from Liofilchem Bacteriology Products (Roseto, Italy). To study the enzymatic activity of the bacteria, the API^®^ ZYM kit, containing the ZYM A + ZYM B reagents (BioMérieux, Marcy-l’Etolie, France), was used. Deionized water was obtained from a Mili-Q water purification system (Millipore, Bedfore, MA, USA). Likewise, methanol was purchased from Fisher Scientific (Madrid, Spain) and acetonitrile from VWR (Leuven, Belgium).

For the polymerase chain reaction (PCR), the “High Pure PCR Template Preparation Kit” from Roche Molecular Systems (Pleasanton, CA, USA) was used. To extract the DNA from the bacteria, ABI PRISM BigDye Terminator Cycle Sequencing Ready Reaction kit and the BigDye Terminator v3.1 cycle drying kit were used, which were acquired from Applied Biosystem Inc. (Foster City, CA, USA), primers 616V and 699R, Taq Dna Polymerase and the five nucleobases were obtained from Thermo Fisher Scientific (Waltham, MA, USA), the “my PCR purification kit” was purchased from Metabion (Gmbh, Germany).

#### 2.1.4. Fish By-Products Broths

Two types of broth were prepared from sea bass: (i) meat (MB) and (ii) by-products (WB), which consisted of a mixture of by-products (37 g of head/100 g of by-products, 29.7 g of guts/100 g of by-products, 13.3g of skin/100 g of by-products and 20g spines/100 g of by-products).

To obtain the broth, the meat was crushed with distilled water, 1:3 ratio (*w/v*), with an OSTER crusher (Albacete, Spain), using the following conditions: 220–240 V, 50/60 Hz and 600 W for 10 min. The mixture was then centrifuged using an Eppendorf 5810R centrifuge (Hamburg, Germany) for 15 min at 4000 rpm. The supernatant was taken in order to obtain a broth without impurities. Then, it was pasteurized in a heat bath at 85 °C for 20 min [19]. Finally, it was centrifuged again in order to obtain a cleaner broth and to facilitate the absence of interferences in the performance of the different tests.

### 2.2. Methods

#### 2.2.1. Isolation and Identification of Bacteria by Morphology, Catalase Test and PCR

To get the bacteria isolated from sea bass, the sample was firstly dissected. The stomach (S), small intestine (I) and colon (C) were separated and grown in a 900 mL MRS Broth liquid medium (LAB selective medium). They were incubated in a culture oven at 37 °C during 48 h under anaerobic conditions using Anaerocult^®^ A.

In order to isolate the different colonies after the incubation, serial dilutions from 10^−2^ to 10^−8^ were made. Then, 100 µL of each dilution were plated in MRS Agar. The plates were incubated at 37 °C for 24 h. 

After the incubation, different colonies were selected. The isolated colonies were placed in a 15 mL tube with MRS Broth medium. A total of 30 bacteria, 7 from the stomach, 7 from the small intestine and 16 from the colon, were isolated. 

In order to determine the morphology of the bacteria, a Gram stain test was performed, and the morphologies were observed with an XJS500 binocular microscope at 40X. The catalase test was performed by mixing an isolated colony in MRS agar with hydrogen peroxide 3%. Moreover, the positive reaction of the catalase test was evaluated by observing bubble formation.

For the identification of the isolated bacteria, they were grown in MRS medium supplemented with 0.05% (*w*/*v*) cysteine and incubated anaerobically at 37 °C from 36 to 72 h. The DNA of the samples was extracted using the “High Pure PCR Template Preparation kit” (Roche), quantified spectrophotometrically and adjusted to a final concentration of 40 ng/µL with ultrapure water, obtained from Sigma-Aldrich (St Louis, MI, USA, USES). To confirm the DNA microarray results, the specific amplification of 16S rRNA gene fragments was performed with “ABI PRISM Big Dye Terminator Cycle Sequencing Ready Reaction kit” from Applied Biosystems Inc. (Foster City, CA, USA) by using the previously described primers (616V: 5′-AGAGTTTGATYMTGGCTCAG-3′ and 699R: 5′-RGGGTTGCGCTCGTT-3′). DNA purity was checked by standard methods.

For 16S rRNA gene amplification, reaction mixtures contained 2 µL (50 pmol/µL) of primers 616V and 699R, 0.5 µL (2U/L) of Taq Polymerase DNA, 10 µL of 10× buffer and 10 µL of dNTP containing 1 mM of dATP, dGTP, dCTP and dTTP (70 µL of miliQ water and 5.5 µL of DNA, with a total of 100 µL). All reagents were purchased from Thermo Fisher Scientific (Waltham, MA, USA).

The DNA template was amplified, with an initial cycle of denaturation at 94 °C for 10 min, followed by 40 cycles of denaturation at 94 °C for 1 min, a second cycle at 55 °C for 1 min for hybridization of the primers, a third 1 min cycle at 72 °C for extension and a final cycle of 72 °C for 10 min. The final sequence was analyzed by individual bands in 2% agarose gels (*w*/*v*) in tris-borate EDTA buffer for one hour at 100 V, then read by electrophoresis. The amplicons were purified using the commercial “mi-PCR purification kit” purchased from Metabion Gmbh (Steinkirchen, Germany) and subsequent sequencing reactions were carried out with the “Big Dye Terminator v3.1 cycle sequencing kit” purchased from Applied Biosystems. The resulting sequences were aligned and compared with the BLASTn online tool. The strain was identified on the highest score.

#### 2.2.2. Enzymatic Activity

The enzymatic activity of the 30 isolated bacteria was studied, using the API^®^ ZYM kit, a semiquantitative method. The results were obtained according to the intensity of the colored reaction, comparing with the table provided by the manufacturer. The results were expressed as nanomoles of hydrolyzed substrate.

To select the strains with the highest proteolytic activity, the trypsin and α-chymotrypsin were selected, taking into account that both are essential for the digestion process, as they are responsible for the degradation of proteins and polypeptides, although others such as cystine arylamidase and valine arylamidase are also proteolytic indicators.

#### 2.2.3. Fermentation of Isolated Bacteria in Meat and By-Products Broths

Once the 7 most proteolytic bacteria were selected, they were inoculated in the different broths (MB and WB) as follows: 1 mL of MRS Broth with bacteria in the exponential growth phase (12 h at 37 °C) was inoculated in 40 mL of the broth. Then, the broths were fermented at 24, 48 and 72 h at 37 °C. A control, using identical conditions, was made for each type of broth.

In order to assess the fermentation process, a bacterial growth study was carried out at 24, 48 and 72 h. Serial dilutions of the different broths were carried out as follows: one hundred microliters of sample were inoculated in a MRS Agar plate to assess bacterial count.

On the other hand, a pH measurement was carried out with the Ph8 + DHS equipment (LabProcess, Badalona, Spain) at different fermentation times, in order to assess the production of organic acids during the fermentation period. The entire procedure was done under sterile conditions in a Telstar MH 100 laminar flow hood (Terrassa, Spain). 

#### 2.2.4. Antifungal Activity

In order to evaluate the antifungal activity of the selected bacteria in the different broths after a 72 h fermentation, an antifungal activity test was performed on solid medium, according to the method described by Varsha et al. [20], with some modifications. The test was carried out against fungi of the genera *Penicillium*, *Fusarium* and *Aspergillus*.

Firstly, the samples were lyophilized in order to concentrate and stabilize them. Then, samples (250 g/L) were resuspended in PDB and filtered using a filter (0.22 µm). The PDA plates, specific for fungal growth, were prepared following the manufacturer’s specifications. To facilitate the growth of the fungi *Penicillium*, *Fusarium* and *Aspergillus*, a cotton swab dipped in water was used.

Once the fungus was inoculated, a total of eight wells were made with the tip of a sterile pipette, in which 100 µL of the resuspended samples were added. The plates were incubated for 48 h at 26 °C. After the incubation, the results demonstrated that the higher halo of inhibition was related to the higher antifungal capacity of the bacteria present in the fermented broth.

In order to determine the Minimum Inhibitory Concentration (MIC), the method previously described by Fothergill [21] was used. The test was performed in a 96-well plate, as follows: a negative control where only PDB medium was added, and secondly, in order to verify that the medium was not contaminated, a positive control consisting of 100 µL of the suspension fungi at 10^5^ spores/mL in PDB, and a volume of 100 μL of sample at final concentration from 0.5 to 250 g/L was used. Immediately, 100 µL of the fungi suspension at 10^5^ spores/mL in PDB was added to all wells, except the negative control. 

All trials were performed in quadruplicate. After 72 h of incubation, the MIC was estimated. The MIC is defined as the lowest concentration of antifungal agent in which there was a visual absence of growth compared to that produced by the growth in the control well. Moreover, in order to confirm the MIC, 10 µL of the concentrations with no growth observed were inoculated in plates with PDA medium, which were incubated in an oven at 26 °C in order to proceed to their visible growth after 72 h. The Minimum Fungicidal Concentration (MFC) was defined as the minimum concentration of an agent promoting a reduction in the number of viable colonies ≥ 99%, with respect to inoculum. All these operations were carried out under sterile conditions in a Telstar MH 100 laminar flow hood (Terrassa, Spain).

### 2.3. Statistical Analysis

The statistical analysis was performed using the InfoStat software^®^ version 2018. All experiments were carried out in triplicate and the differences between the groups were analyzed using a one-way analysis of variance (ANOVA) followed by the Tukey HSD (Honestly Significant Difference) post-test, for multiple comparisons. The level of significance considered was *p* ≤ 0.05.

The correlations were established using the StatAdvisor software^®^ version 2018 and Pearson´s test. The range of correlation coefficients ranges from −1 to +1, and they measure the strength of the linear relationship between the variables. *p*-values ≤ 0.05 indicate correlations significantly different from zero, with a confidence level of 95%.

## 3. Results and Discussion

All isolated bacteria were Gram-positive since the cell wall remained purple after Gram staining, thus indicating that these bacteria were LAB. Moreover, the main form of the cells was coco-bacilli and catalase-negative, these being the most characteristic of LAB [22].

Subsequently, the enzymatic activity of these 30 bacteria was evaluated by using a semi-quantitative method (API^®^ ZYM kit). The activities of 19 enzymes, including alkaline phosphatase, glucosidase and trypsin, were studied, observing great differences according to the fish matrix (stomach, small intestine or colon) studied (Table 1). Bacteria from the stomach had more esterase and alkaline phosphatase activities, while those isolated from the colon had more α-β-glycosidase and n-acetyl-β-glucosaminidase activities. On the other hand, few bacteria had α-mannosidase, α-frucosidase and β-glucuronidase activities. Likewise, it was observed that stomach bacteria had a similar enzymatic activity, since most of them had the same nanomoles value for the same enzymes.

The main aim of this test was the selection of the most proteolytic bacteria to carry out the fermentation tests, in order to maximize protein hydrolysis and the release of small peptides or amino acids. These bacteria were selected taking into account the presence of proteolytic enzymes, trypsin and α-chymotrypsin; therefore, a total of 7 bacteria were selected, four from the stomach, two from the colon and one from the intestine.

**Figure 2:**
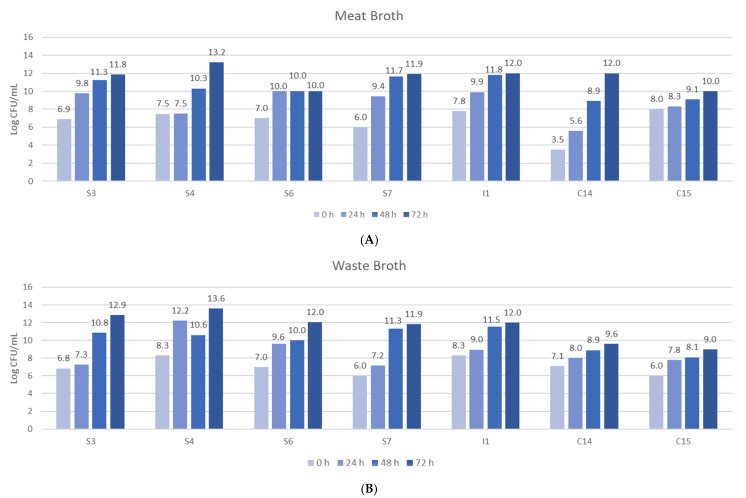
Bacterial growth in (**A**) meat broth (MB) and (**B**) by-products broth (WB) at three fermentation times (24, 48 and 72 h). S: Bacteria from stomach; I: Bacteria from small intestine; C: Bacteria from colon.

Once the seven most proteolytic bacteria were selected, they were fermented in the different culture broths and a bacterial growth test was carried out on a MRS agar plate at different times (24, 48 and 72 h). Figure 2 shows the bacterial growth in meat and by-product broths. 

Regarding the bacterial growth in the by-products broth (WB) after 72 h of incubation, a growth from 6–11, 6–13.6 and 9–12 log CFU/mL occurred for bacteria from the colon, stomach and intestine, respectively. The results demonstrated that stomach bacteria had the highest growth. For meat broth (MB), after 72 h of fermentation, the isolated bacteria from the colon showed a growth from 5 to 12 log CFU/mL, those from the stomach, 6–13.2 log CFU/mL, and those from the intestine, 10–12 log CFU/mL. Thus, in MB, the highest growth was also observed in stomach bacteria.

If the broths are compared, there is no difference regarding bacterial growth, but colon bacteria had a faster growth in the WB, while the gut bacteria grew faster in the MB. No differences in growth may be attributed to a similar nutrient content in the different broths. On the other hand, the bacterial growth was checked and the pH of the broths was evaluated for the same purpose, checking that fermentation was taking place.

**Figure 3:**
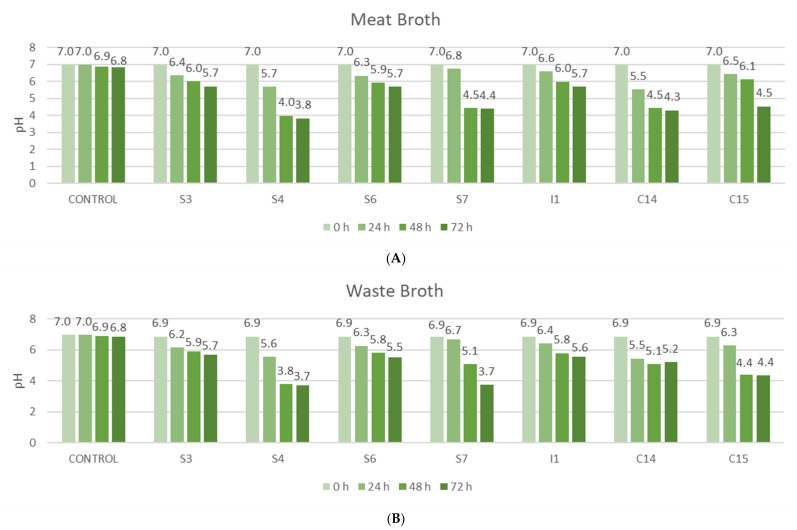
pH values of (**A**) meat broth (MB) and (**B**) by-products broth (WB) at three fermentation times (24, 48 and 72 h). S: Bacteria from stomach; I: Bacteria from small intestine; C: Bacteria from colon.

Figure 3 shows the pH values for the seven bacteria studied. In all strains, it was observed that the pH decreases with the elapse of fermentation period, which means that fermentation occurred, since during the lactic acid fermentation the bacteria convert simple carbohydrates into lactic acid, ethanol and CO_2_, and consequently, pH decreases. Stomach bacteria S4 showed the highest decrease in pH (from 6.9 to 3.7) in the two broths studied. Moreover, the highest decrease of pH in WB (from 6.9 to 3.7) was also shown after using stomach bacteria S7.

Regarding the results obtained about the antifungal activity, strain S3, isolated from the stomach, presented the highest antifungal activity against all fungi, in the different fermented broths, except for *P. verrucosum*, which showed an important resistance to MB (Table 2).

If the results of the broths are observed separately, it is possible to find that the WB produced a greater number of antifungal compounds since strains S3 and S4 showed an important antifungal activity against all the fungi studied. The S7 strain presented an 8 mm inhibition halo against *P. expansum* and the C14 strain for *F. graminearum*. In the MB, strain C14, isolated from the colon, presented an important antifungal activity against the fungi *P. verrucosum* and *F. gramineraum*, while strain S3 had antifungal activity against all the fungi except for *P. verrucosum*. Strains S6, I1 and C15 did not show any antifungal activity for the tested broths (Table 2). 

If the results are compared by fungi, *P. expansum* was the most sensitive fungus, especially with those compounds obtained from WB. On the other hand, *P. verrucosum* was the fungus presenting the highest resistance against all strains (Table 2).

**Figure 4:**
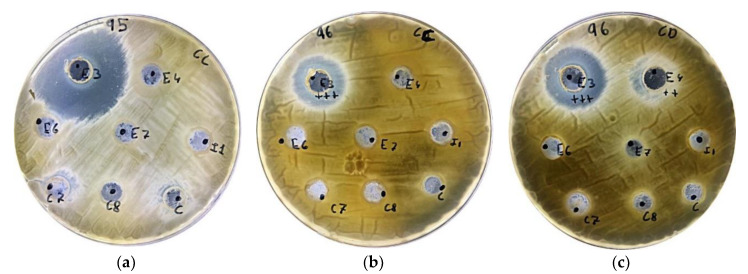
Antifungal activity represented by the halo of inhibition in Petri dishes of: (**a**) fermented MB against *P. expansum*, (**b**) fermented MB against *A. flavus*, and (**c**) fermented WB against *A. parasiticus*.

As in our study, other authors also observed an important antifungal activity of the compounds generated by LABs (*Lactobacillus* strains) against *A. parasiticus*, *A. flavus*, *F. verticilloides* and *P. expansum* [23]. Moreover, in another study with bacteria isolated from gills and stomach, Bajpai et al. [24] also observed that some LAB strains showed an important antimicrobial activity against pathogenic bacteria. In the same line, another study also showed the antifungal activity of 11 LABs strains against *Fusarium oxysporum* [20]. Furthermore, the halo of inhibition presented by strain S3 against fungi *P. expansum* and *A. parasiticus* is also shown in Figure 4.

The tests performed to determine the antifungal activity in liquid medium are shown in Table 3 (bacteria not shown did not have any antifungal activity). The compounds produced by the MB lacked antifungal activity with all strains except for the C14 bacteria, isolated from the colon, which presented an important resistance against *P. verrucosum*, with a MIC value of 16 g/L and a MFC value of 32 g/L. Moreover, C14 bacteria also showed a great ability to delay the growth of *F. graminearum*, with a MIC value of 16 g/L.

Regarding the WB, strain C14 had a MIC of 16 g/L against the fungus *F. graminearum*, although it was not possible to establish a MFC value. As in the test conducted on solid medium, strain S4 gave positive results regarding the growth inhibition for all fungi studied, ranging from 1 g/L for *F. gramineraum* to 32 g/L for *A. flavus*. However, MFC values were only established for *Fusarium* genus. Strain S3 had an antifungal activity against all fungi (1–16 g/L). However, as for strain S4, it was not possible to establish a MFC value for the *Aspergillus* family. On the other hand, strains I1, C15, S6 and S7 did not show either MIC or MFC values.

The results obtained in our study are similar to those obtained by other authors in freshwater fish. So, Ruthu et al. [12] found an inhibitory activity in fermented carp heads. They obtained a MFC value ranging from 60 to 96 mg/ml against the different strains of *Penicillium*. In the same study, the authors also obtained negative results against *A. flavus*. 

The MIC and MFC values are related to the pH values obtained. For instance, the S4 and C14 strains were those presenting more acidic values (3.7 and 4.3, respectively) after 72 h of fermentation. This fact can be attributed to the mechanism of action of LABs against fungi and bacteria, since the accumulation of lactic acid and other organic acids produced by them reduce the pH and consequently, have an inhibitory effect on fungi [25].

In a previous study, it was demonstrated that *L. plantarum* presented an antifungal activity against *Penicillium* in deteriorated citrus and yogurt [26]. Moreover, in the study by Cray et al. [27], *L. plantarum* delayed the growth of *Fusarium culmorum* in oat drink. In contrast, in the study conducted by Ryu et al. [28], a strain of *L. plantarum* isolated from kimchi presented antifungal activity against *Aspergilllus* and *Penicillium* genera fungi. 

Bacteria S3 and S4 were identified by PCR, due to the highest antifungal activity in solid and liquid medium. They were identified as *Lactobacillus plantarum*. According to the studies of Ringo et al. [29], this microorganism was also isolated in Atlantic salmon and Arctic trout [30], pollock [31] and cod [32].

In contrast, there are no previous studies identifying *L. plantarum* in sea bass. It is necessary to emphasize that the intestinal microflora of fish is very variable, and depends on many factors, including environmental conditions and temperature [33], but usually, LABs are the main bacteria in the digestive tract of a healthy fish. According to the study of Nair et al. [34], genera *Carnobacterium* and *Lactobacillus* are the most common bacteria found in the digestive tract of fish. Moreover, Sahnouni et al. [35] also stated that the most common genus found in fish digestive tract is *Lactobacillus*.

One of the main advantages of using *L. plantarum* deals with its use in the industry as a probiotic. For instance, different studies assessed the potential use of fish intestinal tract isolates as probiotics [36]. Moreover, *L. plantarum* is considered as GRAS (Generally Recognized as Safe) and its nutritional applications have been previously evaluated [37]. Since the isolated bacteria are adapted to very low pH, the potential use in humans is considered, but further in vivo research is still needed to verify their application in fish by-products’ valorization to obtain antifungal compounds.

## 4. Conclusions

From the results obtained in this work, it can be concluded that 30 lactic acid bacteria strains were identified and isolated from sea bass gastrointestinal tract. When the most proteolytic strains were used to ferment by-products from the same matrix (sea bass), the fermented extracts obtained presented an antifungal activity against several toxigenic fungi, especially those obtained from sea bass by-products. These results can be useful for the valorization of fish by-products, and also for the development of new natural compounds, which can be used as preservatives in the food industry. However, more studies should be carried out in order to optimize the fermentation process as well as the best way to isolate the targeted antifungal compounds.

## Figures and Tables

**Table 1:** Results of the enzymatic activity of the isolated bacteria (in nmol).

Enzymes	Stomach Bacteria							Small Intestine Bacteria							Colon Bacteria															
	S1	S2	S3	S4	S5	S6	S7	I1	I2	I3	I4	I5	I6	I7	C1	C2	C3	C4	C5	C6	C7	C8	C9	C10	C11	C12	C13	C14	C15	C16
**Alkaline Phosphatase**	10	≥40	≥40	≥40	≥40	5	5	5	5	5	0	30	5	5	20	10	10	20	10	10	10	0	0	0	0	0	10	5	5	0
**Esterase (C4)**	5	20	20	20	5	≥40	≥40	20	10	5	0	20	10	5	0	20	30	20	20	20	20	0	0	0	0	0	0	5	5	5
**Esterase Lipase (C8)**	5	20	10	10	10	20	30	20	30	0	0	10	20	20	0	20	10	10	10	10	10	5	0	0	5	0	5	5	5	5
**Lipase (C14)**	5	0	0	0	0	0	0	0	5	0	0	0	5	0	0	0	0	0	0	0	0	0	0	0	0	0	0	0	0	0
**Leucine Arylamidase**	≥40	30	30	30	20	30	20	0	5	5	0	30	≥40	10	20	30	30	≥40	30	30	20	0	5	0	0	0	≥40	0	10	5
**Valine Arylamidase**	30	5	0	0	0	5	0	5	5	5	0	0	≥40	10	0	5	0	20	5	0	5	0	5	0	0	0	≥40	10	10	5
**Cystine Arylamidase**	20	0	0	10	0	0	10	10	5	0	0	5	10	0	5	10	0	5	0	0	0	10	0	5	0	5	10	10	10	0
**Trypsin**	0	5	0	10	0	10	0	0	5	0	0	0	0	0	0	5	0	0	0	0	0	0	5	0	0	0	5	10	10	5
**α-chymotrypsin**	0	0	10	5	0	0	10	10	0	0	0	5	0	0	0	5	0	0	0	0	0	0	0	0	0	5	0	0	0	0
**Acid Phosphatase**	30	10	10	10	10	5	5	10	5	0	5	5	≥40	10	5	5	0	10	5	10	10	20	5	10	5	10	≥40	30	30	5
**Naphthol-as-bi-phosphohydrolase**	30	10	5	10	20	5	10	10	5	5	5	10	≥40	10	5	10	20	10	5	10	10	10	5	20	10	5	30	30	20	10
**α-Galactosidase**	10	0	0	0	0	0	0	0	5	0	0	0	≥40	0	0	0	0	0	0	5	0	0	0	0	0	0	0	0	0	5
**β-Galactosidase**	30	0	0	0	0	0	0	0	0	0	0	0	≥40	0	0	5	0	0	0	0	0	0	0	0	5	0	≥40	0	0	5
**β-Glucuronidase**	0	0	0	0	0	0	0	0	0	0	0	0	≥40	0	0	5	0	0	0	0	0	0	0	0	0	0	0	0	0	0
**α-Glucosidase**	20	10	10	10	10	5	0	0	0	5	0	0	≥40	0	0	≥40	0	5	10	≥40	30	≥40	≥40	5	0	≥40	10	≥40	≥40	5
**β-Glucosidase**	≥40	10	5	0	10	0	0	5	0	0	0	0	≥40	0	0	30	0	0	20	≥40	30	0	0	30	≥40	0	≥40	0	0	≥40
**N-Acethyl-β-glucosaminidase**	0	0	5	0	0	0	5	0	5	0	0	5	0	10	5	10	20	0	0	0	0	10	5	5	0	0	0	5	0	5
**α-Mannosidase**	0	0	0	0	0	0	0	0	5	0	0	0	0	5	0	0	0	0	0	0	0	0	0	0	0	0	0	0	0	0
**α-Fucosidase**	0	0	0	0	0	0	0	0	0	0	0	0	0	0	0	0	0	0	0	0	0	5	0	0	0	0	0	5	0	0

S: Bacteria isolated from stomach; I: Bacteria isolated from small intestine; C: Bacteria isolated from colon.

**Table 2:** Results of antifungal activity of fermented MB and WB in solid medium.

Fungi	Control	S3	S4	S6	S7	I1	C14	C15
	Meat Broth							
***Aspergillus parasiticus***	-	+++	-	-	-	-	-	-
***Aspergillus flavus***	-	+	-	-	-	-	-	-
***Penicillium expansum***	-	+++	-	-	-	-	-	-
***Penicillium verrucosum***	-	-	-	-	-	-	+	-
***Fusarium graminearum***	-	+++	-	-	-	-	+	-
***Fusarium verticillioides***	-	+++	-	-	-	-	-	-
	**By-Products Broth**							
***Aspergillus parasiticus***	-	+++	++	-	-	-	-	-
***Aspergillus flavus***	-	+	+	-	-	-	-	-
***Penicillium expansum***	-	+++	+++	-	+	-	-	-
***Penicillium verrucosum***	-	+	+	-	-	-	-	-
***Fusarium graminearum***	-	+	+	-	-	-	+	-
***Fusarium verticillioides***	-	+++	+++	-	-	-	-	-

S: Bacteria from stomach; I: Bacteria from small intestine; C: Bacteria from colon. +: <8 mm, ++: 8–10 mm, +++: >10 mm of inhibition halo. “-“: it did not show antifungal activity.

**Table 3:** Minimum Inhibitory Concentration (MIC) and Minimum Fungicide Concentration (MFC).

Fungi	C14		S3		S4		C14	
	MIC	MFC	MIC	MFC	MIC	MFC	MIC	MFC
	Meat Broth		Waste Broth					
***Aspergillus parasiticus***	nd	nd	8	nd	16	nd	nd	nd
***Aspergillus flavus***	nd	nd	16	nd	32	nd	nd	nd
***Penicillum expansum***	nd	nd	1	16	8	16	nd	nd
***Penicillium verrucosum***	16	32	4	8	4	16	nd	nd
***Fusarium graminearum***	16	nd	2	16	1	31	16	nd
***Fusarium verticillioides***	nd	nd	1	8	4	16	nd	nd

S: Bacteria from stomach; C: Bacteria from colon. Results are expressed as g/L. nd: not detected.
